# Complete Genome Sequence and Methylome Analysis of *Bacillus caldolyticus* NEB414

**DOI:** 10.1128/genomeA.01605-17

**Published:** 2018-02-08

**Authors:** Alexey Fomenkov, Tamas Vincze, Fana Mersha, Richard J. Roberts

**Affiliations:** a New England Biolabs, Ipswich, Massachusetts, USA

## Abstract

*Bacillus caldolyticus* NEB414 is the original source strain for the restriction enzyme BclI. Its complete sequence and full methylome were determined using single-molecule real-time sequencing.

## GENOME ANNOUNCEMENT

The bacterial strain *Bacillus caldolyticus* NEB414 was originally isolated as an extreme thermophile organism and is now housed in the New England Biolabs culture collection. It is the original source of the type II restriction enzyme BclI, which recognizes and cleaves the DNA sequence T↓GATCA, producing 5′ cohesive ends compatible for cloning DNA digested with Sau3AI (↓GATC), BamHI (G↓GATCC), or BglII (A↓GATCT) (1).

Genomic DNA from a culture of *B. caldolyticus* NEB414 was purified using a modified Qiagen method, and the genome was sequenced using the Pacific Biosciences (PacBio) RS II sequencing platform. Briefly, SMRTbell libraries were constructed from a genomic DNA sample sheared to an average size of ~10 to 20 kb using the G-tubes protocol (Covaris, Woburn, MA, USA), end repaired, and ligated to hairpin adapters. Incompletely formed SMRTbell templates were digested with a combination of exonuclease III and exonuclease VII (New England Biolabs, Ipswich, MA, USA). Qualification and quantification of genomic DNA fragments and SMRTbell libraries were performed using a Qubit fluorimeter (Invitrogen, Eugene, OR, USA) and a 2100 Bioanalyzer (Agilent, Santa Clara, CA, USA). One 12-kb SMRTbell library was prepared according to the modified PacBio sample preparation protocols, including additional separation with the BluePippin system, and sequenced using C4-P6 chemistry and 3 single-molecule real-time (SMRT) cells with a 240-min collection time. Sequencing reads were processed, mapped, and assembled by the Pacific Biosciences SMRT analysis pipeline using the HGAP3 protocol and polished using Quiver (2) to yield 943.9 Mb of sequence data, which was assembled into two contigs, a closed circular genome of 3,457,580 bp with 159.9-fold coverage and a plasmid, pBcl1, of 28,002 bp with 362.8-fold coverage. The assembled sequences were annotated using the NCBI Prokaryotic Genome Annotation Pipeline (PGAP) and have been deposited at DDBJ/EMBL/GenBank (accession numbers CP025074 and CP025075).

The advantage of the PacBio sequencing platform is its ability to detect the epigenetic state of sequenced DNA, which allows for the identification of modified nucleotides and their corresponding motifs. Epigenetic modification at each nucleotide position was measured as kinetic variations (KVs) in the nucleotide incorporation rates, and methylated motifs were deduced from the KV data (3–5). Four DNA methyltransferase recognition motifs were detected, each containing m6A modifications. The motifs were then matched with methyltransferase genes in the genome, and the results are shown in Table 1. They have also been deposited in REBASE (6).

**TABLE 1:** Summary of methyltransferases identified in *B. caldolyticus* NEB414

Motif^a^	Assigned name	Methylation type	Restriction modification type
TG**AT**CA	M.Bcl	m6A	II alpha
CG**A**NNNNNNN**T**ARC	M.BclII	m6A	I gamma
GCC**A**T	M.BclIII	m6A	III beta
GAG**A**G	M.BclIV	m6A	III beta

a Modified bases are shown in bold; they are underlined when on the complementary strand.

### Accession number(s).

The complete genome and pBclI plasmid sequences of the *B. caldolyticus* isolate reported here have been deposited in GenBank under the accession numbers CP025074 and CP025075.
